# Remarks on Goitre

**Published:** 1825-01

**Authors:** Thomas D. Drug


### Art. IV.—*Remarks on Goitre.* By ~~Thomas D. Drug~~, M.D.

In June, 1821, I became a resident at Matlock Bath, in Derbyshire, and remained there upwards of two years. I soon discovered, from the prevalence of bronchocele in the neighbourhood, that it had, with much propriety, been considered by writers as endemical; and that a fair opportunity might be afforded me of entering on an investigation of the remote causes of that extraordinary affection, (the knowledge of which has ever been a desideratum,) and likewise of ascertaining the effects of iodine in it as a curative mean. As many facts were subsequently elicited confirmatory of conclusions drawn, I feel much confidence in making the present communication.

In the vicinity of Matlock there exist various factories, particularly the extensive cotton-mills of Messrs. Arkwright. In this district, goitre is so very common, as to make me feel no hesitation in averring that, among the lower classes of females who have attained the twentieth year, a proportion of at least twelve in twenty are, in one degree or another, the subjects of it; it often runs through families, and is commonly denominated *the thick neck;* and is by no means an unfrequent occurrence in the other sex. Goitre may be there seen of all the intermediate sizes, from a pigeon's egg to the magnitude of an infant's head; and distressing symptoms occasionally occur, requiring depletion, even in instances of moderate enlargement, from pressure on the internal jugulars and larynx,—such as cephalalgia, vertigo, dyspnœa, sense of suffocation, &c.

In my attempts to discover the remote causes, I was naturally induced, in the first place, to make inquiry concerning the prevailing opinions of the county, by which I discovered that the same errors therein existed as elsewhere,—*e. g.* water impregnated with calcareous matter, or other mineral principle; exertion in ascending hills; and moist atmosphere of the valley.

Now the first, as a cause, I apprehend, does not obtain, because the complaint prevails in situations where water from gritstone, and other sources, is in use, equally as where it proceeds from calcareous strata. As to the latter, they can only concomitantly act as exciting causes, and merely by inducing in the system a certain degree of relaxation and augmented circulation of blood: effects not peculiar to that county, but to every other mountainous district.

Not having obtained from this source any satisfactory information, determined me to institute an inquiry into the general customs of the lower orders of inhabitants, and more particularly those who were the subjects of the complaint: the following facts, as relating to diet, and appearing to be of paramount importance, are the result:—The poor are bad managers in regard to selection and plain cookery; their food consisting chiefly of [illegible] oaten cake, milk, tea, and inferior potatoes, or other vegetable matter, and hardly ever conjoined with animal food of any description. The cakes are prepared of oatmeal made into a thin paste with water, and then fermented with leaven (a substitute for barm), which, after being half-baked and still soft, are piled up, not unfrequently in the bed-chamber;* and, either to save trouble, or that they may improve in quality, a sufficient number are made to last five or seven days; at which period, it is true, they increase in acerbity, if that be considered an advantage. This custom is no doubt of remote antiquity; and, during the late war, the use of this species of food, in consequence of the high price of wheat, was almost universal among the lower classes; many of whom, if asked why they do not eat wheaten bread, now it can readily be obtained, in preference to sour cake? reply, "Because the latter is the strongest and best food." It is true some, from habit, may prefer it; but I am satisfied that a large majority would choose wheaten bread, did they consider it equally thrifty: and the explanation which it strikes me may be given to the general supposition of sour cake being a stronger or more thrifty food, is that arising from its indigestible quality, and, as a consequence, remaining longer on the stomach; thereby preventing that natural or more speedy return of appetite, as instanced in quicker digestion. Even simple oaten cake, as made in Scotland and other parts of the united kingdom, is heating, and of an indigestible quality; but, with the addition of leaven, its action on the system appears in a manner specific, or at least more decided. If my recollection serve me, calculous and cutaneous disorders were very frequent in Edinburgh, perhaps arising, in a great measure, from the quantity of oaten food consumed.

From conversations with several of the faculty in Derbyshire, also numerous persons with and without goitre, I have learnt that nephralgic, cutaneous, and obstinately dyspeptic cases, are exceedingly frequent in many parts of the county.

Now, upon a consideration of the indigestible and acescent quality of leavened oaten cake, are we not brought to the conclusion, that to it must be attributed a considerable degree of mischief in the human system? And, if I apprehend rightly, milk, from its acescent tendency when taken with it, may rather heighten than diminish its noxious effects.† From habit, the stomach may, to a certain extent, accommodate itself to any kind of aliment; but, when we take into consideration the con-

---

* A certain portion of the fermented mass, being left in the tub, becomes leaven, or what is called by the natives "old Doshan;" and, from custom, I am informed the troughs, in many instances, remain uncleansed for five, or even seven years.

† I have been consulted in many gastric cases, where strong marks of structural deviation existed, evidently the sequelæ of long-continued impaired function, kept up by a constant application of some exciting cause unknown to the patient. A respectable practitioner in Derbyshire with candour acknowledged that he had never been able to account for the obstinacy of dyspepsia, before the sour cake was suggested as a cause.

tinued use of such ingesta as those in question, (being little varied, morning, noon, and night,) corresponding effects must naturally be supposed to accrue.*

I have, by argument, brought over a number of persons to this view of the subject; and some, under an assurance that medicine would avail them little without a change in their accustomed diet, have yielded: but I fear the great bulk will not be readily persuaded to relinquish a habit which has been so long established, although so evidently founded on error.

It may not be improper here to observe, that, although my experience is confined to Derbyshire, I feel a firm conviction that the causes of endemic goitre are every where analogous in their nature and *modus agendi.* Thus, if it be true that sour cake, by acting as a pernicious ferment, either alone or combined with other vegetable matter, exerts a powerful influence in the production of goitre and other disease, may we not infer, upon the same principle of reasoning, that bad acescent cider, as a common beverage, may, *cæteris paribus*, produce similar results?

In Herefordshire, the lower classes drink a very bad description of cider; and, in Switzerland, rye-bread is eaten, half-baked, which no doubt, from the absence of yeast, is made with some leaven; and, probably, the inhabitants are in the daily use of some acescent beverage, and other improper ingesta.†

Though fully satisfied of the influence of this fermentative principle in the production of goitre, yet, as regards a rationale, I must profess my incompetency. Has sympathy between the thyroid system and a faulty state of the chylopoietic viscera, any share in the morbid concatenation? or does it depend upon a vitiated state of the general system, or both conjoined? The disorder certainly appears to be connected with imperfect digestion and assimilation; and, this being allowed, I beg to submit whether it be possible, under such circumstances, that perfect nutrition, and a healthy state of the solids, can be the result? Perhaps this view of the subject may, after further researches,

---

* I have heard of many instances where, after several years' disuse of the cake, an accidental return to it has been the cause of surfeit, attended by a troublesome eruption on the skin.

† The following extract is from the letter of a clerical friend, then residing in Switzerland:—"But, with respect to goitre and your questions, I have made every inquiry that my circumscribed connexion will allow; and the only issue of my research is—the Faculty know very little about the matter. I find, however, that the proportion of males affected, in comparison of females, is great, but not defined; that the food of the sufferers consists of rye-bread and other food badly cooked,—i. e. half baked; that gastric diseases are common among them. In my journey in that part called the Valais, I found the goitres to be larger and more numerous; and in this, as in other parts where goitres were more prevalent, I found that idiotism was more common."

lead us not only to a knowledge of the proximate causes of goitre, but of the share the thyroid gland (so abundantly supplied with nerves and blood-vessels) has in the economy of the system.

Bronchocele and scrofula have been imagined, by some writers, and not without reason, to be strongly allied. May not their essence be the same,—only that the former is brought into existence by the co-operation of some local or accidental cause? The variation in point of character and termination may, perhaps, arise from a peculiarity in structure and function of the thyroid gland. The correctness of this idea seems strongly manifested by the frequency of scrofula in its usual forms in this country, and particularly in the synchronous existence of goitre with enlarged jugular and cervical glands, so often discovered in the same person.

In respect to *ratio medendi*, my objects were, in the first place, to correct, by means of mild alteratives and aperients, the state of the digestive organs; advising, almost as a *sine qua non*, the disuse of sour cake and all acescent drinks, and the substitution of a more nutritious diet, with a proportion of flesh-meat; secondly, to attempt the cure of the local affection by the internal exhibition of iodine. The tincture, the only form employed, was given in mint-water twice or thrice a-day, in augmented doses, according to age, and not exceeding in any instance fifteen drops. The cases under treatment were between the ages of fourteen and twenty-two, and, although not numerous, sufficed to convince me of the value of this remedy,—at least, in endemic goitre; six of the slighter cases being cured within a month, and several others, though in a progressive state of recovery, were left in *statu quo*, simply from a dislike formed by the patients to the medicine.

A further inquiry regarding the preceding particulars, must not only prove interesting as relates to science, but of great practical utility, and will, I trust, be pursued by those better qualified, and now more favourably situated, than myself; and with that degree of candour which should ever be inseparable from the medical profession, and indeed every other branch of science, when truth is the object of inquiry.

From the mass of evidence already obtained may be deduced the following corollaries, as applicable to the county of Derby:

1. Goitre, in certain districts, is an endemic affection; and it is evident that water, employed for domestic purposes, does not exhibit any specific or other influence in the production of it.

2. The constant use of an acetous ferment, as an article of food, appears, under certain favourable circumstances, to be an exciting cause of bronchocele; such circumstances consisting either in a scrofulous diathesis, or a peculiar state of system, the result of imperfect nutrition, confinement to factories, &c. &c.; and goitre exists in a precise ratio with their degree of operation.

3. It is not confined to females, but is seldom, if ever, met with in the male sex, unless the person has been reared on sour cake and other unwholesome food.

4. Idiotcy is by no means uncommon, and seems connected with an imperfect development of the system: hence the old adage, "*Mens sana est in corpore sano.*"

5. Goitre is sometimes, though rarely, congenital.

6. Fish, flesh, and other nutritious articles of food, counteract, in a considerable degree, the injurious effects of acetous ferment: thus, to the disuse of them much mischief may be attributed.

7. Endemic goitre does not extend to the higher circles, wherein the disease occurs only in solitary instances, as in other counties in which it is not endemic, and is then characterised by a strong strumous diathesis. The same obtains with poor families who settle in Derbyshire, provided they do not adopt the peculiar customs of the county.

8. The tincture of iodine may be considered a pretty certain remedy for endemic goitre in young subjects. It evidently exerts a powerful action on the lymphatic system, and, by carefully regulating the doses and mode of exhibition, may be employed without danger to life.

