# Erratum in Our Last Number

**Published:** 1825-02

**Authors:** 


Erratum *in our last Number.*

P. 49, for "Thomas D. Drug," read William D. Dray.

