# Errata in Dr. W. D. Dray's Paper, No. 311

**Published:** 1825-03

**Authors:** 


Errata in Dr. W. D. Dray's Paper, No. 311.

Page 50, 6 lines from bottom, *dele* "some."

— 51, 5 lines from bottom, insert an ? after "system."

— 53, top line, for "causes" read "cause."

